# Functional metabolomics: from biomarker discovery to metabolome reprogramming

**DOI:** 10.1007/s13238-015-0185-x

**Published:** 2015-07-02

**Authors:** Bo Peng, Hui Li, Xuan-Xian Peng

**Affiliations:** Center for Proteomics and Metabolomics, State Key Laboratory of Biocontrol, School of Life Sciences, MOE Key Lab Aquat Food Safety, School of Life Sciences, Sun Yat-sen University, Guangzhou, 510275 China; Molecular Foundry, Lawrence Berkeley National Laboratory, Berkeley, CA 94720 USA

**Keywords:** metabolomics, discovery metabolomics, reprogramming metabolomics, metabolic strategy, metabolic regulation

## Abstract

Metabolomics is emerging as a powerful tool for studying metabolic processes, identifying crucial biomarkers responsible for metabolic characteristics and revealing metabolic mechanisms, which construct the content of discovery metabolomics. The crucial biomarkers can be used to reprogram a metabolome, leading to an aimed metabolic strategy to cope with alteration of internal and external environments, naming reprogramming metabolomics here. The striking feature on the similarity of the basic metabolic pathways and components among vastly different species makes the reprogramming metabolomics possible when the engineered metabolites play biological roles in cellular activity as a substrate of enzymes and a regulator to other molecules including proteins. The reprogramming metabolomics approach can be used to clarify metabolic mechanisms of responding to changed internal and external environmental factors and to establish a framework to develop targeted tools for dealing with the changes such as controlling and/or preventing infection with pathogens and enhancing host immunity against pathogens. This review introduces the current state and trends of discovery metabolomics and reprogramming metabolomics and highlights the importance of reprogramming metabolomics.

## INTRODUCTION

Metabolism is the cornerstone of life, with the set of life-sustaining chemical transformations within the cells of living organisms to provide building blocks and energy equivalents for fundamental biological processes, such as cell growth, cell differentiation and environmental adaptation. The chemical transformations, one chemical being transformed into a new chemical, are organized as metabolic cascades, which are catalyzed by a series of enzymes. As the major players in metabolic pathways, enzymes drive the desirable reactions but also regulate metabolic pathways positively or negatively to maintain the energy homeostasis within the cells. Regulation of metabolism entails several levels of controls including transcriptional, posttranscriptional and allosteric mechanisms to eventually affect enzyme abundance and kinetics (Fuhrer and Zamboni, 2015), indicating metabolism is a complex and dynamic process with systemic outcomes represented by metabolites. The thorough and comprehensive analysis of the metabolites, named as metabolomics, would provide the insights of cellular activities without considering the change of genetic or epigenetic changes.

Metabolomics has been extensively used in basic and applied research in recent years and gradually become a mutually complementary technique to genomics, transcriptomics and proteomics (Dumas, 2012; Castro-Santos et al., 2015). Metabolomics specifically addresses the activity of the small molecules(<10 kDa) produced by active and living cells during their life cycles, which are not accessible by genomics, transcriptomics and proteomics (Peng, 2013). Therefore, metabolomics can give an instantaneous snapshot of the physiological status of the cells, which tells people how the metabolic profile of a complex biological system is changed in response to stress like adverse factors, environmental alterations or physiological adaption to dietary change (Patel and Ahmed, 2015; Zhang et al., 2014; Gibbons et al., 2015). Metabolomics speeds up the understanding of global metabolic characteristics, elucidation of metabolic mechanisms and identification of metabolic biomarkers. A striking feature of metabolism is the similarity of the basic metabolic pathways and metabolites among vastly different species, while genes, mRNAs and proteins are diversified from species to species. In this regard, metabolomics is a universal language to describe a complex life activity across different species. For example, the set of carboxylic acids that are best known as the intermediates in the TCA cycle are present in all known organisms, being found in species as diverse as from unicellular bacterium like *Escherichia coli* to huge multicellular organisms like elephants and complex multicellular organisms like humans. These similarities in metabolic pathways and metabolites are likely due to their early appearance in evolution and their retention because of their efficacy. Compared to other OMICs, metabolomics provides broad scope and yet direct information on the integrated cellular response to changes in internal and external environment with low demand in material and sample preparation (Fuhrer and Zamboni, 2015). While the current state and trends of high throughput metabolomics profiling focus on the purpose of discovering biomarkers and hunting for metabolic mechanism, a prospective direction, namely reprogramming metabolomics, highlights the way to use metabolomics approach for the purpose of prevention and treatment of disease through reconstitution of perturbed metabolic pathways (Peng et al., 2015a). Therefore, metabolomics is not only used to discover the metabolome of cells or organisms under different conditions but also used to evaluate the reprogramming efficacy of exogenous metabolite treatment, which were termed as discovery metabolomics and reprogramming metabolomics, respectively. In this review, we summarize the current state and trends of discovery metabolomics and reprogramming metabolomics and highlight the importance of reprogramming metabolomics for therapeutic purpose.

## TECHNIQUES FOR DISCOVERY METABOLOMICS

Metabolomics is a universally applicable, comprehensive analytical approach for the identification and quantification of metabolites in a biological system, leading to description and understanding of a metabolic-responsive profile (Holmes et al., 2008; Suhre et al., 2011). The discovery metabolomics has been established and applied as the method of choice for investigating metabolic phenotypes in life science research. Metabolomic studies consist of two sequential procedures: (1) High throughput detection of an all-inclusive spectrum of the complete profile of low-molecular weight metabolites in a biological sample; (2) Model recognizable analysis for interpreting detected data.

To identify, quantify and characterize the small molecules/metabolites in cell or organism, metabolomics primarily depends on high throughput analytical technologies. The most commonly used technologies in metabolomics are nuclear magnetic resonance (NMR) spectroscopy and mass spectrometry (MS). The most popular analytical methods in MS are gas chromatography-mass spectrometry (GC-MS), liquid chromatography-mass spectrometry (LC-MS) and capillary electrophoresis coupled to mass spectrometry (CE-MS). These four analytical methods provide technical pillars for high throughput discovery metabolomics. Each of these techniques has its own advantages and disadvantages. NMR provides an *in situ* way for monitoring certain classes of metabolites with high reproducibility and less inter-laboratory variation. More importantly, NMR allows a rapid, nondestructive and automated analysis of crude extracts, and the quantitative detection of many different groups of metabolites. But the drawback is the limitation of sensitivity and dynamic range (Wolfender et al., 2015). GC-MS integrates the advantage of high resolution, separation reproducibility and the stable metabolite fragmentation patterns from the standardized MS electron ionization energy of 70 eV and is ideally suited for the analysis of both volatile and nonvolatile compounds (or compounds with volatile derivatives) (Gao and Xu, 2015). GC-MS has long been used for identifying metabolites of biological fluids. However, the variety of metabolites that can be analyzed with this technique is limited by the volatility or the ability to form appropriate volatile derivatives of the molecules. LC-MS allows the analysis of thermally labile but non-volatile compounds. LC-MS detects the molecules that range from polar sugars and non-aromatic organic acids to various lipids (Commisso et al., 2013). Recently, CE-MS has emerged as an attractive technique for the profiling of polar and charged compounds in biological samples and used in metabolomics due to complementation of unique chemical information that is not provided by GC-MS and LC-MS (Ramautar et al., 2015). The combination of GC-MS with NMR or/and LC-MS has widely used in separation, dramatization and characterization of small molecules/metabolites (Kumar et al, 2014; Gao and Xu, 2015). This leads to the improvement of structural analysis and better interpretation of targeted metabolome.

Most of the metabolomics data is recorded as unassigned peaks of different intensity on different retention times, masses or mass fragments (mass spectrometric data) or chemical shifts (NMR data). Analysis and interpretation of these data show more common features among different MS-based platforms. The area of each compound peak is extracted from the total ion chromatograms with software. Single ions are used to deconvolute overlapping peaks. Compounds are identified based on matching with retention time and mass spectrum. The data matrix must be normalized before statistical analysis. The normalization factors are dependent on different requirements. The internal standards or statistical models derived from the complete dataset, e.g., the total peak intensities, the quartile range or the corresponding metabolite fragments are generally used in the relevant quality control samples (Gao and Xu, 2015). After normalization, multivariate statistical methods or pattern recognition methods are used to handle the acquired data, to search for the discriminating features and to identify the biomarkers from the tested samples.

## DISCOVERY METABOLOMICS

The metabolome can be a very sensitive measure of an organism’s phenotype because metabolites are the final products of enormous genome-wide or proteome-wide interactions. This fact has made metabolomics particularly useful in the study of environment-gene and -protein interactions (Rojo et al., 2015; Wang et al., 2015; Zhang et al., 2015), the identification of disease biomarkers (Peng et al., 2015b), revelation of pathogenesis (Kim et al., 2015; Ghartey et al., 2015) and the discovery of novel drugs (Mastrangelo et al., 2014; Krug and Müller, 2014). Metabolism is also embedded in the sensing and signaling machinery, integrating information transmitted from internal and external environment of cells (Helms et al, 2015; Zheng et al., 2015). Therefore, metabolism is pivotal in decision-making in both health and disease and a valuable target for targeted therapy. Current metabolomics studies essentially focus on what can be known from the metabolome. The discovery metabolomics studies aim to measure the up-regulation and down-regulation of metabolites in abundance, and the impact of their variations on metabolic pathways and networks. Typically, the discovery metabolomics is addressed to identify biomarkers to improve early diagnosis, accurate prognosis and aid adequate monitoring of disease and understand physiological changes in response to environmental alteration (Gibbons et al, 2015).

Recently, the metabolomics approach has been widely used in investigation of cellular activity. In biomedical application, this approach is usually employed to identify altered abundance of metabolites and the affected metabolic pathways in a disease-specific manner for use as diagnostic markers or therapeutic targets (Budczies et al., 2015). Budczies et al., used GC-MS based metabolomics to quantify the dysregulation of the glutamate-glutamine equilibrium and characterize glutamate accumulation as new diagnostic opportunity in breast cancer. These findings may have important implications for patient stratification related to utilization of glutaminase inhibitors (Budczies et al., 2015). Metabolomics is also performed to characterize the interactions of organisms with their environment for the discovery of new disease risk biomarkers and diagnostics. Changes in the metabolic profiles in a group of 389 healthy smelter workers exposed to lead, cadmium and arsenic were studied on pooled serum samples using proton nuclear magnetic resonance spectroscopy. These multivariate metabolomics datasets were analyzed with principal component analysis and partial least squares discriminant analysis. Analysis of metabolic profiles of people exposed to heavy metals suggested that energy metabolism disturbance could be induced by heavy metals (Dudka et al., 2014). Changes in lipid fraction, unsaturated lipids and in the level of amino acids suggested the perturbation of lipids metabolism and amino acids metabolism. This approach is capable of identifying intermediate biomarkers of response to toxicants at environmental/occupational concentrations, paving the way to its use in a monitoring of smelter workers exposed to low doses of lead, cadmium and arsenic (Dudka et al., 2014). Do et al., presented a generic, data-driven network-based approach for structuring and visualizing metabolite correlations within and between multiple body fluids including blood, urine and saliva, enabling unbiased interpretation of metabolomics multifluid data (Do et al., 2015).

In preclinical and clinical drug development, the applications of metabolomics have a considerable scope in the pharmaceutical industry, almost at each step from drug discovery to clinical development. These include determination of drug target, potential safety and efficacy biomarkers and mechanisms of drug action, the validation of preclinical experimental models against human disease profiles, and the discovery of clinical safety and efficacy biomarkers (Kumar et al., 2014). Farag et al., indicated comparative metabolomics could be utilized for Senna drug quality control analysis. They revealed secondary metabolite compositional differences among Senna species through utilizing both UPLC-MS and NMR and recognition methods (Farag et al., 2015). Weckmann et al., showed that a single injection of Ketamine had an impact on the major energy metabolism pathways and indicated that 2-ketoisovalerate, glutathione, maleate, methylmalonate, fumarate and cytosine qualify as biomarkers for the Ketamine drug response (Weckmann et al., 2014). Since treatment of mammalian cells with chemotherapeutic drugs can result in perturbations of nucleotide pools, Liu et al., monitored these perturbations in cultured human tumor cells for a better understanding of the mechanism of action of these drugs. They showed that GMP and ATP were the best potential biomarkers for DNA-damaging drugs, as well as GMP, ATP and UDP for the antimetabolite and the mitotic spindle agents. These results provide a preliminary evidence for the role of pharmacometabolomics in the preclinical development of drugs (Liu et al., 2014).

Applications of discovery metabolomics in nutritional research can include three main areas: identification of dietary biomarkers, study of diet-related diseases and identification of biomarkers of disease and application to dietary intervention studies as a tool to identify molecular mechanisms (Gibbons et al., 2015). Metabolomics is impactful on all of these areas and contributes significantly on the study of diet-health relationships (Gibbons et al., 2015; Brennan, 2014). Wagner et al., showed that the nutritional state of a freshwater invertebrate, *Daphnia magna*, could be determined by analyzing endogenous metabolites using hydrogen nuclear magnetic resonance-based metabolomics. In addition, the metabolite composition of *D. magna* was correlated to the diets they were taking and environmental stresses they suffered. Thus, dietary-specific induced changes in metabolite composition of animal consumers hold considerable promise as indicators of nutritional stress and will be invaluable for future studies of animal nutrition (Wagner et al., 2015). The Mediterranean diet is a dietary pattern with beneficial effects on human health. Vazquez-Fresno et al., used NMR-based metabolomics to assess the effect of an Mediterranean diet on urinary metabolome and revealed that the most prominent hallmarks concerning Mediterranean diet groups were related to the metabolism of carbohydrates, creatine, creatinine, amino acids, lipids and microbial cometabolites. The application of NMR-based metabolomics enables the classification of individuals regarding their dietary pattern and highlights the potential of this approach for evaluating changes in the urinary metabolome at different time points of follow-up in response to specific dietary interventions (Vázquez-Fresno et al. 2014). Floegel et al., investigated the associations between diet, physical activity, cardiorespiratory fitness and obesity with serum metabolite networks in a population-based study and found that amino acids were particularly positively associated with cardiorespiratory fitness and physical activity. C6-sugar and acylcarnitines were positively associated with obesity and inversely associated to the intake of whole-grain bread. Phospholipids were negatively associated with obesity and coffee intake. Metabolic networks of coffee intake and obesity were inversely correlated. Strong positive correlation was observed between metabolite networks of body mass index and waist circumference, as well as the metabolic networks of cake and cookie intake with cardiorespiratory fitness and intake of whole-grain bread. Therefore, lifestyle factors and phenotypes seem to interrelate in various metabolic pathways (Floegel et al., 2014).

Development of stress-tolerant plants and reducing the gap between the pace of natural products research and highly-demanded modern drug discovery are emerging topics in current plant industry. The applications of discovery metabolomics greatly benefit the elucidation of molecular mechanisms involved in biotic or abiotic stress, phytomedicines quality control analysis and drug discovery from natural sources (Nakabayashi and Saito, 2015; Jorge et al., 2015; Mahrous and Farag et al., 2015). Salt stress is an important factor that limits crop production worldwide. Kusuda et al., compared the metabolic profiles between myo-inositol 3-phosphate synthase-overexpressing and wild-type rice and found myo-inositol 3-phosphate synthase improved salt stress tolerance through activation of central metabolism, such as glycolysis, the pentose phosphate pathway, the TCA cycle and inositol metabolism (Kusuda et al., 2015). Sun et al., used NMR techniques to examine the metabolic responses of maize plants grown under different conditions including soil drought, soil salinity, heat and multiple concurrent stresses. Drought-stressed maize plants subjected to salt or heat stress showed distinct integrated metabolic profiles compared with those exposed to either stress alone. Moreover, the TCA cycle and core nitrogen metabolism were identified, which were related to their multiple functions during plant growth. The primary metabolic responses to soil drought, heat and combined drought and heat stress occurred in a time-dependent manner, indicating that plasticity at the metabolic level might allow maize plants to accumulate their metabolic ranges in response to environmental change (Sun et al., 2015).

In microbiological research and industrial fermentation applications, the discovery metabolmics is used in metabolic flux analysis, gene function elucidation, enzyme function analysis and fermentation optimization (Gao and Xu, 2015). NtcA, a conserved cAMP receptor protein-type transcription factor among cyanobacteria, regulates gene expression in response to nitrogen status. Osanai et al., overexpressed *ntcA* in *Synechocystis sp*. PCC 6803, which dramatically affected sugar, purine/pyrimidine nucleotide, organic acid and amino acid metabolisms. This study set an example to advance the understanding of metabolic regulation of unicellular cyanobacteria (Osanai et al., 2014). Dörries et al., investigated the impact of antibiotic compounds with different cellular targets on the metabolome of *Staphylococcus aureus* HG001. They found far-ranging effects within the metabolome by comparing the metabolic profiles of unstressed and stressed staphylococcal cells in a time-dependent manner. They showed that ciprofloxacin altered the pool of deoxy- nucleotides and peptidoglycan precursors; erythromycin tended to increase the amounts of intermediates of the pentose phosphate pathway and lysine; fosfomycin inhibited the first enzymatic step of peptidoglycan synthesis; vancomycin and ampicillin inhibited the last stage of peptidoglycan construction on the outer cell surface. Each antibiotic also affected intracellular levels of the TCA intermediates (Dörries et al., 2014). These results suggested an association of antibiotic action with metabolic environment. Wu et al., used GC-MS approach to disclose the metabolic mechanism manipulated by glycine, which led to higher antifungal activity, in a fungal strain *Penicillium citrinum* W1 isolated from a Southwest Indian Ocean sediment sample. They showed that elevated glycine, serine and threonine metabolism promoted biosynthesis of fatty acids and the TCA cycle and thereby contributed to bacterial growth and antifungal activity. Further the replacement of glycine with serine demonstrated the reliability of the identified glycine, serine and threonine metabolism. These results indicate functional metabolimcs is a powerful tool for understanding mechanisms through supplementing compound (Wu et al., 2015).

Recently, the metabolite-based genome-wide association study (mGWAS) has allowed the simultaneous analysis of the genetic and environmental impacts on homeostasis (Adamski, 2012). It has emerged as a powerful alternative forward genetics strategy to dissect the genetic and biochemical basis of metabolism, providing a useful approach for functional gene identification with more precision, standardization, robustness and sensitivity (Adamski and Suhre, 2013). Feng et al., performed an mGWAS on stools from advanced adenoma and carcinoma patients and from healthy subjects, revealing microbial genes, strains and functions enriched in each group and suggested that fecal microbiome-based strategies might be useful for early diagnosis and treatment of colorectal adenoma or carcinoma (Feng et al., 2015). The developed mGWAS described the profile that metabolites and small molecules were not independent. They were organized in biochemical pathways and in a wider metabolic network, which was self-dependent on various genetic and signaling networks for its regulation (Dumas, 2012). Importantly, a new generation of integrative study of genomics, transcriptomics, proteomics and metabolomics improves an understanding of the response mechanism of organisms to internal and external environments (Dumas et al., 2014; Bielecka et al., 2014; Klähn et al., 2015).

## REPROGRAMMING METABOLOMICS

Metabolomics is a powerful tool for exploring the alterations in the metabolite abundance, and metabolic pathways and networks which are involved in various pathophysiological conditions and offers the platform for identification of genotype-phenotype as well as genotype-envirotype interactions (Kumar et al., 2014). On the other hand, the similarity of the basic metabolic pathways and components between different species as well as the commercially available metabolite lead to a possibility that metabolome can be reprogrammed by exogenous metabolites (Peng et al., 2015a), which is very difficult to be achieved by conventional genetic means. Metabolome is the collection of end products of cellular activities, reflecting the status of cells at certain stage. Cells resistant to certain factor can be sensitized to the factor by administrating certain metabolite. Therefore, metabolomic approach is used not only to screen biomarkers of diseases, but also to identify crucial metabolites or potential drugs to revert or enhance the status according to requirement. This is because the metabolites have biological activities (Matsuda et al., 2014; Carey et al., 2015). The binding of various types of metabolites with hormones, fatty acids, drugs or other xenobiotics, transport proteins and receptors proteins has been reported (Matsuda et al., 2014; Tannahill et al., 2013). The binding of metabolites to proteins leading to conformational and functional change of the protein is of growing interest recently (Matsuda et al., 2014). Thus, metabolomics change is a strategy that host mounts to cope with changes of the internal and external environment. Exogenous crucial metabolites can reprogram the metabolic strategy to adapt or suppress such changes.

Metabolites play biological roles in cell activity as a substrate of enzymes and a regulator to other molecules including proteins and nucleic acids. Metabolite abundance is decisive to the biological roles. When the abundance is changed, activity of the enzymes and action of the regulated molecules will be modulated, which affect the metabolic pathways and networks and possibly modulate the metabolic flow. Differential activation or suppression of one or more metabolic pathways could be a critical feature of the response (stress or disease) phenotype. If a responsive ‘metabolome’ could be identified, it may be possible to induce susceptibility or resistance to response-induced treatment by exogenous administration of a crucial metabolite that is less or more abundant in the response metabolome. In other words, the response-resistant or -susceptible metabolome could be converted into the response-susceptible or resistant metabolome by engineering the metabolic environment, respectively. This leads to metabolome reprogramming. The reprogramming metabolomics approach can be used to clarify the mechanism of cell response and then based on the mechanism to establish a framework to develop convenient and efficient tools for dealing with the changes such as controlling and/or preventing infection with pathogens and enhancing host immunity against pathogens.

**Figure 1:**
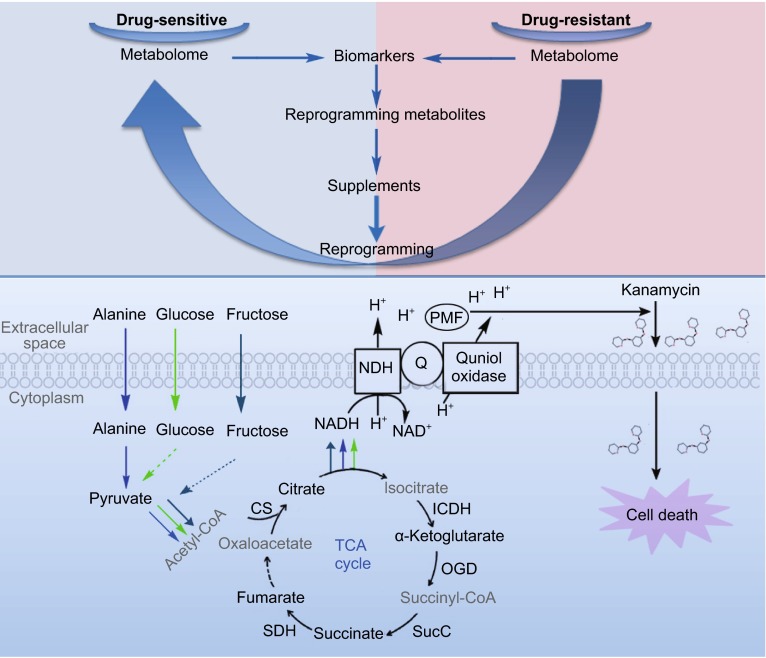
**Antibiotic-resistant metabolome is reprogrammed to antibiotic-susceptible metabolome by exogenous alanine, fructose and glucose**. These exogenous metabolites promote TCA flux, increase proton-motive force and lead to kanamycin uptake

Bacterial antibiotic resistance is a threat to human health and to the viability of animal and plant species in the human food chain (Li et al., 2012; Lin et al., 2014). However, novel drugs that can manage infections by multidrug-resistant bacteria have proved elusive. We demonstrated that the bacterial susceptibility to antibiotics was strongly associated with metabolic states, and that specific metabolic profiles were correlating with certain antibiotic-resistance. Glucose, fructose and alanine abundances are greatly suppressed in kanamycin-resistant *Edwardsiella tarda* revealed by GC/MS based metabolomics. Exogenous alanine, glucose or fructose restores susceptibility of multidrug-resistant *E. tarda* to killing by kanamycin. Similar results were obtained with other Gram-negative bacteria including *Vibrio parahaemolyticus*, *Klebsiella pneumoniae*, *Pseudomonas aeruginosa* and Gram-positive bacterium *Staphylococcus aureus.* These results were also reproduced in a mouse model for urinary tract infection. Therefore, metabolites that reverted the metabolomics profile of an antibiotic-resistant strain to that of an antibiotic-sensitive strain could potentially revert the antibiotic resistance (Fig. 1) (Peng et al., 2015a; Su et al., 2015). The study proposes a novel approach to identify metabolic modulator through investigation of metabolomics, by which crucial modulators can be used for therapeutic purpose through reprogramming metabolome (Bhargava and Collins, 2015).

Elimination of microbes through regulation of immunity has been widely accepted. This leads to widespread use of immune modulators to control infections. Interferon (IFN) family is a large group of cytokines involved in innate immune response against various microorganisms. However, whether IFN functions in antimicrobial property by metabolic pathways is largely unknown. When GC/MS-based metabolomics was applied to humoral fluid of zebrafish (*Dario rerio*) exposed to three different doses of IFN-α2b, designed as IFN-L (low dose), IFN-M (middle dose) and IFN-H (high dose), forty-three IFN-responsive metabolites were identified. Analysis on the dose-related metabolites indicated that biosynthesis of unsaturated fatty acids was enriched only in IFN-M and IFN-H, which was related to IFN-α2b high protection against bacterial infection. Moreover, exogenous fatty acids, especially unsaturated linoleic acid, elevated survival ability of zebrafish infected with extracellular pathogenic *V. alginolyticus* and intracellular pathogenic *Edwardsiella tarda.* Since the dose of IFN-α2b was proportional to protective ability against bacteria and exogenous fatty acids played the similar roles in eliminating microbial infection, activation of biosynthesis of unsaturated fatty acids became a downstream pathway to mediate anti-microbial function of IFN-α2b. These results reveal an unknown mechanism by which IFN-α2b protects host from microbial infections, highlighting the ways to understand action of IFN in content of metabolic regulation (Zhao et al., 2014).

**Figure 2:**
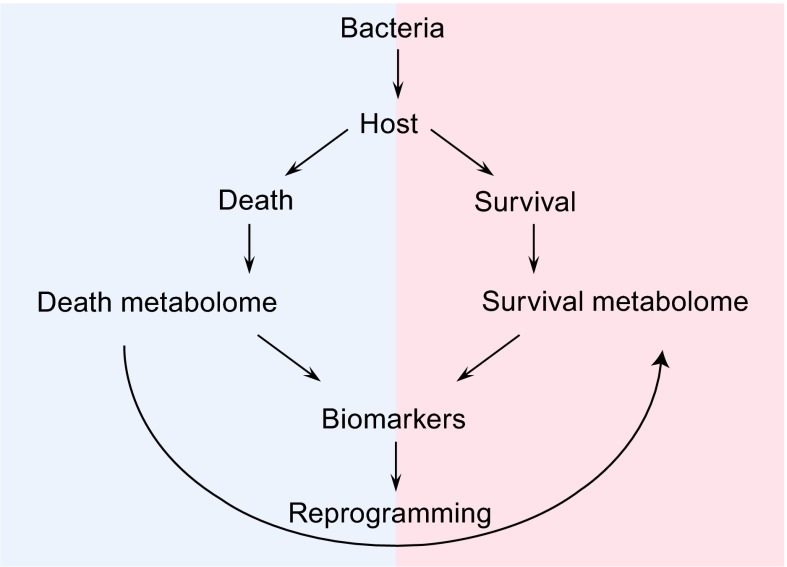
**Bacterial infection-sensitive metabolome** **is reprogrammed to bacterial anti-infective metabolome by exogenous crucial metabolites**. Sublethal dose of pathogen causes hosts either to die or survival, which is related to their metabolomes. Crucial metabolites can be identified by comparison of the two metabolomes. Exogenous addition of the crucial metabolites increases the chances of survival

Besides the reprogramming metabolome of immune regulator such as IFN-α2b, the approach can be used to restore host defense ability from susceptibility to resistance to pathogens. Streptococcosis causes massive tilapia kills, which results in heavy economic losses of tilapia farming industry. Current antibiotic options are limited and possess severe practical limitations and potential adverse environmental impacts. GC-MS based metabolomics is used to characterize variation of metabolomes in response to *S. iniae*. Nine key metabolites that separate the survivals from the dying and control were identified. N-acetylglucosamine was the most critical metabolite elevated in survival groups but decreased in dying groups. The reversal change formed a characteristic feature as a result of tilapias against infection caused by *S. iniae*. When exogenous N-acetylglucosamine was added, the survival ability significantly elevated by at least 35%. In anti-*S. agalactiae* metabolome, significant decrease of several amino acids and their metabolic pathways was characterized. L-proline was identified as a crucial biomarker. Three-day L-proline-supplemented tilapias by intraperitoneal injection or oral administration before challenging with *S. agalactiae* showed the significant higher resistance against the challenging bacteria, elevating 60% survival rates in group with 2 mg L-proline injection (Cheng et al., 2014). In the same way, the approach can be used to reprogram a metabolic strategy to modulate the effect of temperature on bacterial toxicity. *S. agalactiae* killed more tilapias in higher than lower temperatures, where 50% mortality and 70% mortality were reported for tilapias cultured in 25°C and 30°C, respectively while no death was detected at 20°C (Zhao et al., 2015). We investigated the metabolome change in the livers of tilapia. Thirty-six and forty-five varied abundance of metabolites were identified in livers of tilapias cultured at 25°C and 30°C, respectively. Decreasing abundance of L-proline was identified as a crucial biomarker for indexing higher water temperature and a potential modulator to reduce the high death. This was validated by engineering injection or oral addition of L-proline. Exogenous L-proline led to elevated amino acid metabolism, which contributed to the elevated survivals (Zhao et al., 2015). These results highlight the way to identify drug potentials to combat microbial infections though reprogramming metabolome (Fig. 2).

## METHODOLOGIES FOR REPROGRAMMING METABOLOMICS

**Figure 3:**
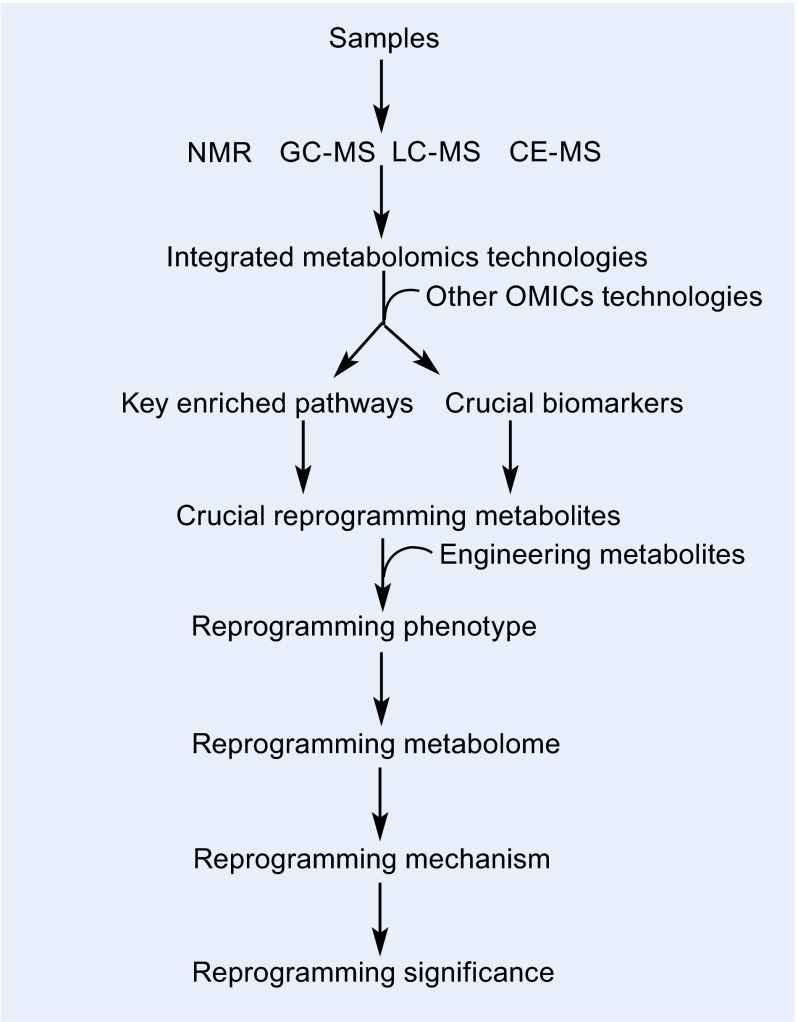
**Outline of reprogramming metabolome**

Reprogramming metabolomics identifies crucial biomarkers and pathways while discovery metabolomics does to provide potential metabolic modulators, reprogramming metabolites, for modulating an existing metabolome to a targeted metabolome. This leads to reconstitution of metabolic strategy. The reconstitution can be demonstrated by metabolomics analysis and functional assays (Peng et al., 2015a). The metabolomics analysis is used to validate the effect of exogenous metabolite boosting on varied metabolites and pathways and identify the metabolic flux magnitudes into each metabolite pool by determining the mass isotopomer distribution for all labeled compounds (Peng et al., 2015a). The functional assays provide proofs on whether the reconstituted metabolic strategy may show the similar phenotypes and the mechanism of action (Peng et al., 2015a; Su et al., 2015; Cheng et al., 2014; Zhao et al., 2014; Ma et al., 2015). Thus, reprogramming metabolomics reveal the response and reconstituted mechanisms by analysis of the response metabolome and reconstituted metabolome, respectively, in which methods on molecular biology and biochemistry, cell biology and immunology will become basic tool to elucidate these mechanisms. The general methodologies of the reprogramming metabolomics are outlined in Fig. 3.

## SUMMARY AND PERSPECTIVES

While recent studies on metabolic profiles, metabolic biomarkers and metabolic mechanisms by the discovery metabolomics have substantially increased our understanding of cellular activity, it has also become clear that we should pay more attention to the reprogramming metabolomics. The striking feature on the similarity of the basic metabolic pathways and components between vastly different species makes the reprogramming metabolomics possible, forming a characteristic feature that differentiates from other OMICs. The reprogramming metabolomics combines response metabolome with reconstitution metabolome together to clarify response and reconstitution mechanisms and provide potential drugs for the purposes of disease prevention and treatment. Future research towards the theory and application of reprogramming metabolomics for the reconstitution of host metabolic strategies or the modulating of pathogenic microbes’ adverse metabolomes needs to be focused on understanding of the mechanisms underlying metabolic strategy, identification of crucial biomarkers that can be efficient for reprogramming metabolome, and disclosing of the reprogramming mechanisms. While gene-to-metabolite and protein-to-metabolite analysis by mGWAS and integrated OMICs provides a useful tool for improvement of crucial biomarker identification and mechanism understanding, the regulation of metabolites to other molecules including proteins and nucleic acids and the clarification of metabolic fluxes by exogenous metabolites are a useful clue to understanding the reprogramming mechanisms.

The reprogramming metabolomics described in this paper has so far only been ‘the beginning’, but the approach has proven to be useful tools for reconstitution of an aimed metabolome and understanding of the reconstitution mechanisms, such as reverting ‘antibiotic-resistant metabolome’ to ‘antibiotic-susceptible metabolome’ by promoting the TCA cycle, NADH and proton motive force and thereby increasing antibiotic uptake (Peng et al., 2015a; Su et al., 2015). Particular attention should be paid to identify highly efficient reprogramming metabolites that result in reinforcing host’s metabolic strategy against adverse factors and reverting microbes’ resistant-metabolomes to susceptible-metabolomes to killing. The identification should be based on understanding of metabolic profile, clarification of metabolic pathways and network, and disclosing of metabolic mechanisms. Furthermore, the synergistic use of reprogramming compounds can elevate then reprogramming effects (Peng et al., 2015a), which highlights the way to boost the reprogramming output. A further factor that may be neglected is the clarification of metabolic fluxes caused by exogenous reprogramming metabolites. The metabolic fluxes play a crucial role in reconstitution of the reinforcing or reverting metabolomes, determination of the reconstitution effect and understanding of the reprogramming mechanisms. Finally, the goal to be pursued in the future will be to transfer these approaches to clinical use by identifying efficient reprogramming metabolites as drugs. Since metabolites are generally non-poisonous to organisms, the use of the reprogramming drugs becomes relatively simple, which brings the gospel to human heath, animal feed and aquaculture. Therefore, the reprogramming metabolomics will provide an alternative way for nutrition care, physical recovery, disease prevention and treatment.
